# The emerging role of photon-counting detector CT: primary experience on the integrated assessment of acute knee injuries

**DOI:** 10.1186/s41747-025-00616-8

**Published:** 2025-08-09

**Authors:** Frank M. Zijta, Alexander Truyens, Rene E. Weijers, Joachim E. Wildberger, Pieter J. Emans, Thomas Flohr

**Affiliations:** 1 Department of Radiology and Nuclear Medicine, Maastricht University Medical Centre (MUMC+), Maastricht, The Netherlands; 2 CARIM School for Cardiovascular Diseases, Maastricht University, Maastricht, The Netherlands; 3 CAPHRI Care and Public Health Research Institute, Maastricht University, Maastricht, The Netherlands; 4 Department of Orthopedic Surgery, Joint-Preserving Clinic, Maastricht University Medical Centre (MUMC+), Maastricht, The Netherlands; 5 Faculty of Health, Medicine and Life Sciences (FHML), Maastricht University (UM), Maastricht, The Netherlands

**Keywords:** Anterior cruciate ligament, Computed tomography, Image processing, Knee injuries, Magnetic resonance imaging

## Abstract

**Abstract:**

Early accurate diagnosis of osseous and soft tissue injuries following acute knee trauma is crucial for guiding clinical management and preventing chronic instability. Radiography is the appropriate first imaging test applied to detect traumatic osseous injuries. CT is indicated based on clinical symptoms and radiographic concordance. In this acute phase, soft tissue injuries are often clinically overlooked due to swelling and restricted motion, which significantly limit comprehensive physical examination. Moreover, both x-ray and conventional CT imaging are insufficient for addressing this issue due to their limited soft tissue contrast resolution. If clinical suspicion of soft tissue injury persists, an MRI will be performed at a later stage. This may lead to undesirable delays in diagnosis and treatment, thereby potentially impacting patient outcomes. Photon-counting detector CT (PCD-CT) offers enhanced, integrated diagnostic possibilities. The use of spectral imaging data, including color-coded virtual non-calcium (VNCa) images, enables the detection of bone marrow edema (BME) and visualization of key stabilizing soft tissue structures, which may assist emergency department clinicians in determining initial treatment, follow-up, and the need for additional imaging. This technical note illustrates the integral use of ultra-high resolution spectral PCD-CT in a case of a knee injury following an alpine skiing accident.

**Relevance statement:**

The integration of photon-counting detector computed tomography with spectral imaging in acute knee trauma enhances visualization of osseous and soft tissue structures, improving diagnostic accuracy. It may optimize early triage and guide initial treatment for soft tissue injuries.

**Key Points:**

- Photon-counting detector CT (PCD-CT) enables comprehensive fracture, edema, and soft tissue assessment.
- Case-based notable correlation between injuries suspected on color-coded spectral imaging and MRI.
- Photon-counting detector CT (PCD-CT) may enhance early clinical decision-making in knee trauma.

**Graphical Abstract:**

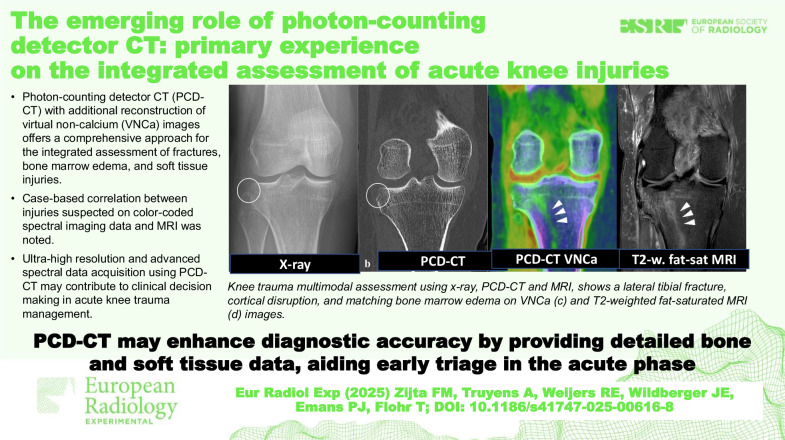

## Background

Conventional radiography is the initial imaging modality performed in the context of acute knee trauma, mainly to detect fractures and osseous avulsion, but also to recognize indirect signs of soft tissue injury [1]. However, it has certain limitations, necessitating additional cross-sectional diagnostic imaging in case of an occult fracture, or if traumatic soft tissue injury or cartilaginous pathology of the knee is suspected.

For accurate tissue characterization of acute knee injuries, magnetic resonance imaging (MRI) has generally been recognized as the reference standard [2, 3]. MRI provides multiplanar imaging with a superior soft-tissue contrast resolution, allowing the evaluation of traumatic osseous injuries, bone marrow edema (BME), cartilage, and ligamentous injuries [4]. Yet, MRI is not always available and may be contraindicated in certain patients, urging the need for alternative diagnostic imaging strategies.

CT, typically used for detailed assessment in complex extremity injuries, has technologically evolved to offer additional diagnostic benefits. Dual-energy CT (DECT) with conventional energy-integrating detectors (EID) has emerged as a promising tool in acute musculoskeletal (MSK) imaging [5, 6]. Nevertheless, it has not found its way into clinical routine, as the acquisition of dual-energy data requires special scan modes with limitations in most EID-CT systems, *e.g*., in terms of maximum field of view, insufficient optimization of radiation dose, artifacts and limited spatial resolution.

More recently, the use of a new generation CT with photon-counting detectors (PCD-CT) has increasingly been evaluated [7]. PCD-CT offers advantages for MSK imaging beyond conventional EID-CT: improved soft tissue contrasts due to higher signal contribution from low-energy x-ray, higher spatial resolution up to 40 line pairs/cm without radiation dose penalty due to smaller detector elements without separation layers, and ubiquitous spectral information for tissue differentiation and material-specific contrasts [8]. The combination of ultra-high-resolution (UHR) morphological imaging and spectral tissue differentiation with a single CT scan was previously unavailable and might transform diagnostic accuracy and clinical utility.

In this technical note, we present the use of ultra-high resolution (UHR) spectral PCD-CT for the integrated assessment of fractures, BME, and soft tissue injuries in acute knee trauma. Enhanced PCD-CT might have potential clinical utility in the early triage of soft tissue injury detection.

## Data acquisition and image evaluation

CT scans were performed on a first-generation dual-source PCD-CT (NAEOTOM Alpha, software version VB10; Siemens Healthineers AG). Scan data were acquired in a dual-source spectral UHR mode (QuantumPeak, collimation 96 × 0.2 mm), with one x-ray tube operated at 70 kV and the other at 150 kV with a tin filter (Sn 150 kV) for improved spectral separation [9]. The gantry rotation time was 0.25 s. The image quality level was set to 75 with anatomical dose modulation (CAREDose4D, Siemens) turned on. Both UHR images (reconstructed slice-thickness 0.2 mm, kernel Br80) and spectral post-processing (SPP) images (reconstructed slice-thickness 0.8 mm, kernel Qr44) were reconstructed from the same UHR scan data. Br80 with a limiting resolution of 28.0 line pairs/cm was chosen as a good compromise between the highest resolution and low image noise [10]. Qr44, with a lower limiting resolution of 11.2 line pairs/cm, was selected to optimize the low-contrast resolution in the spectral SPP images. The SPP images carry low- and high-energy images for further spectral evaluation in their image header: low-energy images are beam hardening-corrected virtual monoenergetic images (VMI) at 53 keV reconstructed from the 4-energy bin data measured at 70 kV, high-energy images are polychromatic T3D images (mean energy 95 keV) reconstructed from the Sn150 kV data. The detailed scan and reconstruction parameters are listed in Table S1.

**Fig. 1:**
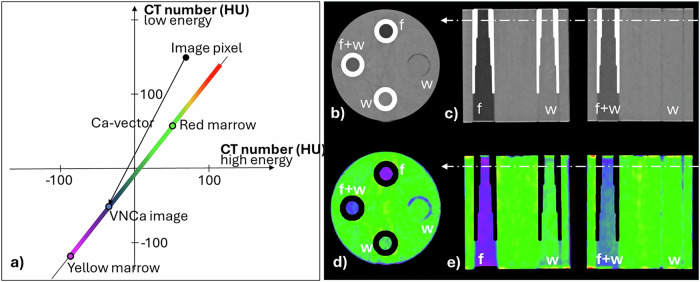
**a** Schematic representation of the image-based calculation of a VNCa image. The plot represents CT numbers (measured in Hounsfield Units, HU) at low energy on the vertical axis and at high energy on the horizontal axis. Mixtures of yellow and red bone marrow lie on a straight line in this CT number space. Calcium content is removed from each image pixel by projection onto this line along the Ca-vector. In the VNCa image, only yellow and red bone marrow (or edema) remain, their composition mapped along the mixture line and visually represented through a color-coded gradient. **b**, **c**, **d**, and **e** Images of a phantom with cylindrical rods with a hydroxyapatite shell, filled with water-equivalent material (w), fat-equivalent material (**f**), and a 50%/50% mixture of fat and water (f + w). Top row: VMIs at 70 keV; bottom row: VNCa images with the Ca-removed and remaining materials color-coded. **b**, **d** Axial images at a z-position indicated by the dashed line. **c**, **e** MPRs along the *z-*direction

To visualize both bone-marrow edema (BME) and soft tissue injuries, an image-based technique, which has already been established in dual energy CT with EID [11], was adapted to PCD-CT (Bone Marrow application, syngo.via, VB80, Siemens). Figure 1a illustrates the basis of this process. The coordinates of a single pixel are determined by the measured HU numbers of that pixel at high imaging energy (the *x*-axis) and the measured HU numbers at low imaging energy (the *y*-axis). In the absence of image noise, mixtures of yellow and red bone marrow lie on a straight line defined by yellow bone marrow (CT_low_ = -115 HU, CT_high_ = -85 HU at 70 kV/Sn 150 kV) and red bone marrow (CT_low_ = 55 HU, CT _high_ = 51 HU). To isolate the marrow components, calcium content in an image pixel is removed by projecting its CT_low_/CT_high_ numbers onto the bone marrow line along the calcium vector (Ca-vector; slope 1.9 at 70 kV/Sn 150 kV), resulting in a virtual non-calcium (VNCa) image (see Fig. 1a). The VNCa image is color-coded, with yellow bone marrow displayed in violet, red bone marrow in green, and tissues with higher CT-number in red. BME appears as a focal green area in a violet-blue environment of yellow bone marrow. Soft tissue structures, *e.g*., ligaments and tendons, appear yellow-red; their visualization benefits from the suppressed bone signal in the VNCa images.

## Phantom test

The Bone Marrow application was tested with a phantom (QRM Quality Assurance in Radiology and Medicine GmbH), consisting of a water-equivalent cylinder (10 cm in diameter) with four removable cylindrical rods (2 cm in diameter), one made of water-equivalent material, the other three with outer shells of hydroxyapatite with varying thicknesses along the *z*-direction (1 mm- 2 mm- 3 mm) to simulate cortical bone. These three cylinders are filled with fat-equivalent material (corresponding to yellow bone marrow), water-equivalent material (simulating red bone marrow or edema), and a 50%/50% mixture of fat and water.

The phantom was scanned with the scan mode described above. Parameters of the Bone Marrow application were modified to account for the measured CT-numbers of the fat-equivalent (CT_low_ = -110 HU, CT_high_ = -65 HU) and water-equivalent materials (CT_low_ = 0 HU, CT_high_ = 0 HU), which differ from the actual yellow- and red bone marrow.

VMIs of the phantom at 70 keV are shown in Fig. 1b, c, and VNCa images in Fig. 1d, e—the VNCa images demonstrate the correct removal of the hydroxyapatite shell, and the expected color coding of the 4 cylinders according to their filling (water-equivalent in green, fat-equivalent in violet, 50%/50% mixture in blue-green). We observed only a slight dependence of the results on the thickness of the enclosing hydroxyapatite shell (Fig. 1e), which suggests reasonably stable clinical results in variable bone environments.

## Case report

A 50-year-old female patient presented to the emergency department because of a swollen and painful right knee, one day after an alpine skiing accident. Clinical examination revealed significant knee effusion and restricted range of motion due to pain. Significant valgus instability under valgus stress at 20° flexion (International Knee Documentation Committee (IKDC) score C-abnormal) was demonstrated at the right knee, indicating a moderate to severe medial collateral ligament (MCL) injury. The varus stress test at 20° flexion suggested an intact lateral collateral ligament (IKDC score A-normal). The patient reported an immediate inability to bear weight following the trauma.

**Fig. 2:**
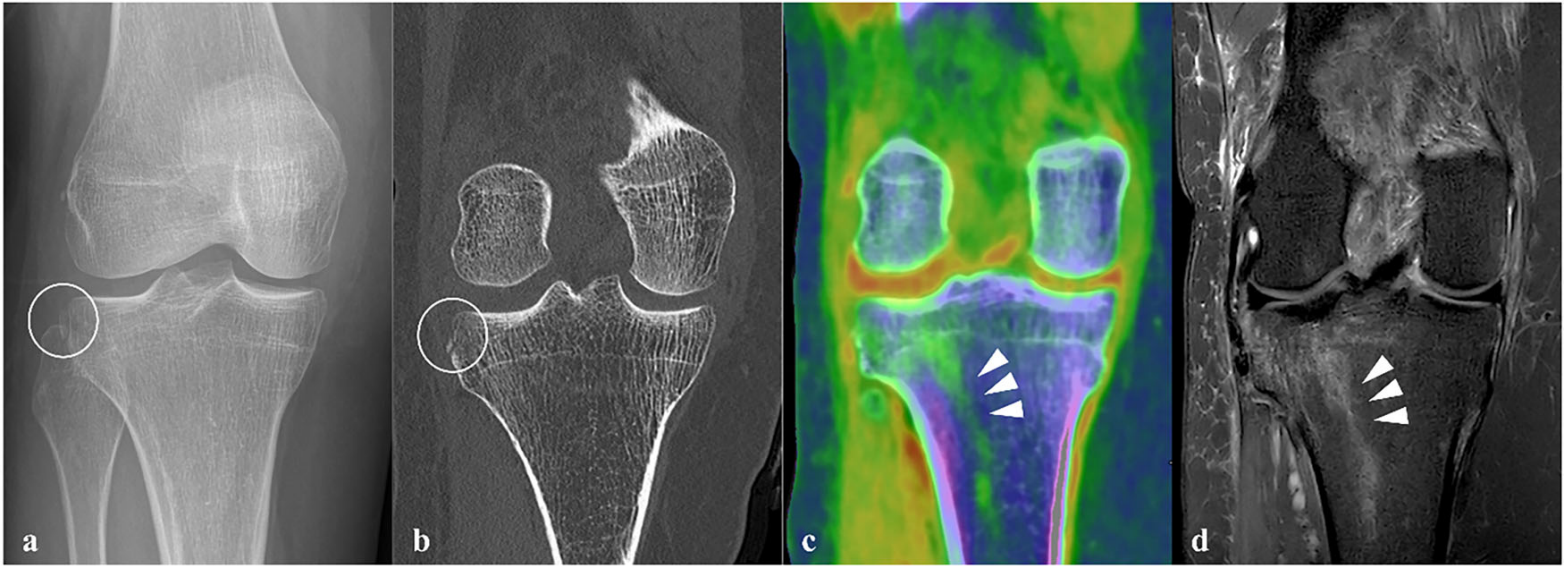
Multimodal imaging assessment of acute trauma of the knee, using radiography, PCD-CT (VNCa image), and MRI. **a** Knee x-ray (AP-view) shows a discrete Segond fracture (circle) of the lateral tibial plateau. **b** Native high-resolution PCD-CT (coronal view) depicts the subtle cortical discontinuity (circle) at the lateral aspect of the tibial plateau without additional distinct fracture lines. **c** Coronal color-coded VNCa image displays linear-oriented bone marrow edema (*green*, arrowheads) in the lateral tibial plateau, extending into the distal and central tibial metaphysis. **d** Corresponding coronal T2-weighted fat-saturated MRI image illustrates bone marrow edema, with a similar distribution to the VNCa image

Conventional, two-directional radiography was performed, revealing a suprapatellar effusion and a subtle osseous fragment adjacent to the lateral proximal tibia, consistent with a Segond fracture (Fig. 2a). Given the subtlety of the osseous abnormalities and the suspicion of fracture extension into the lateral tibial plateau on radiography, additional PCD-CT was performed to better characterize the extent of bony injury.

**Fig. 3:**
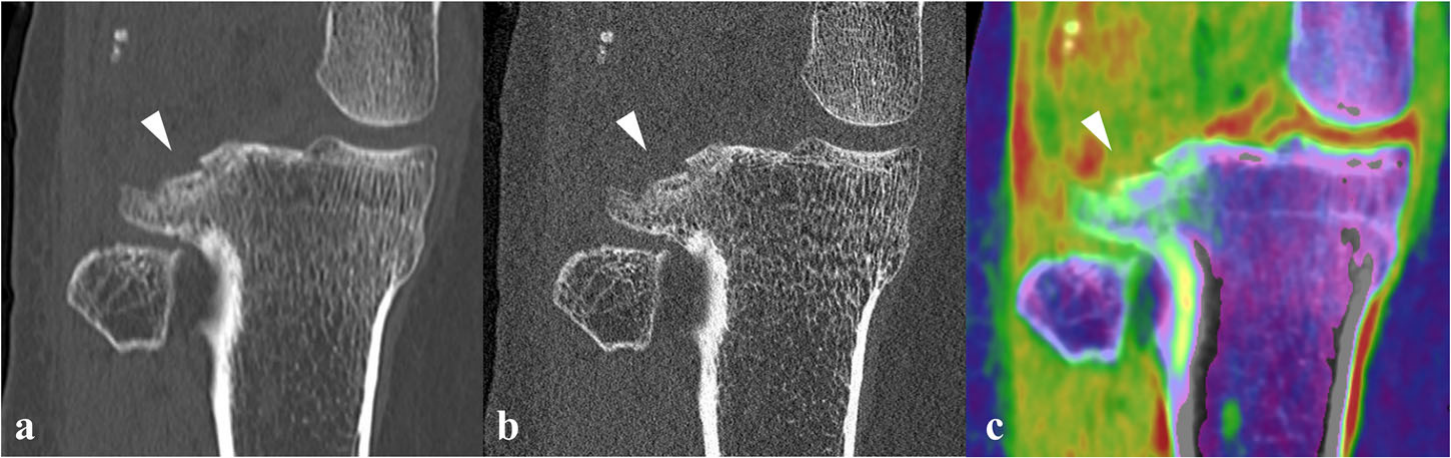
PCD-CT of the lateral tibial plateau using standard reconstruction, ultra-high-resolution (UHR), and color-coded virtual non-calcium (VNCa) display. **a** Coronal standard-resolution image (slice thickness 1.0 mm, kernel Br64) shows a fracture at the posterior side of the lateral tibial plateau with slight depression (arrowhead). **b** Corresponding ultra-high-resolution (UHR) image (slice thickness 0.2 mm, kernel Br80) enhances fracture conspicuity by better delineating the cortical discontinuity (arrowhead). **c** A spectral post-processed VNCa image reveals perifocal bone marrow edema (green, arrowhead) extending from the fracture site into the lateral tibial plateau

**Fig. 4:**
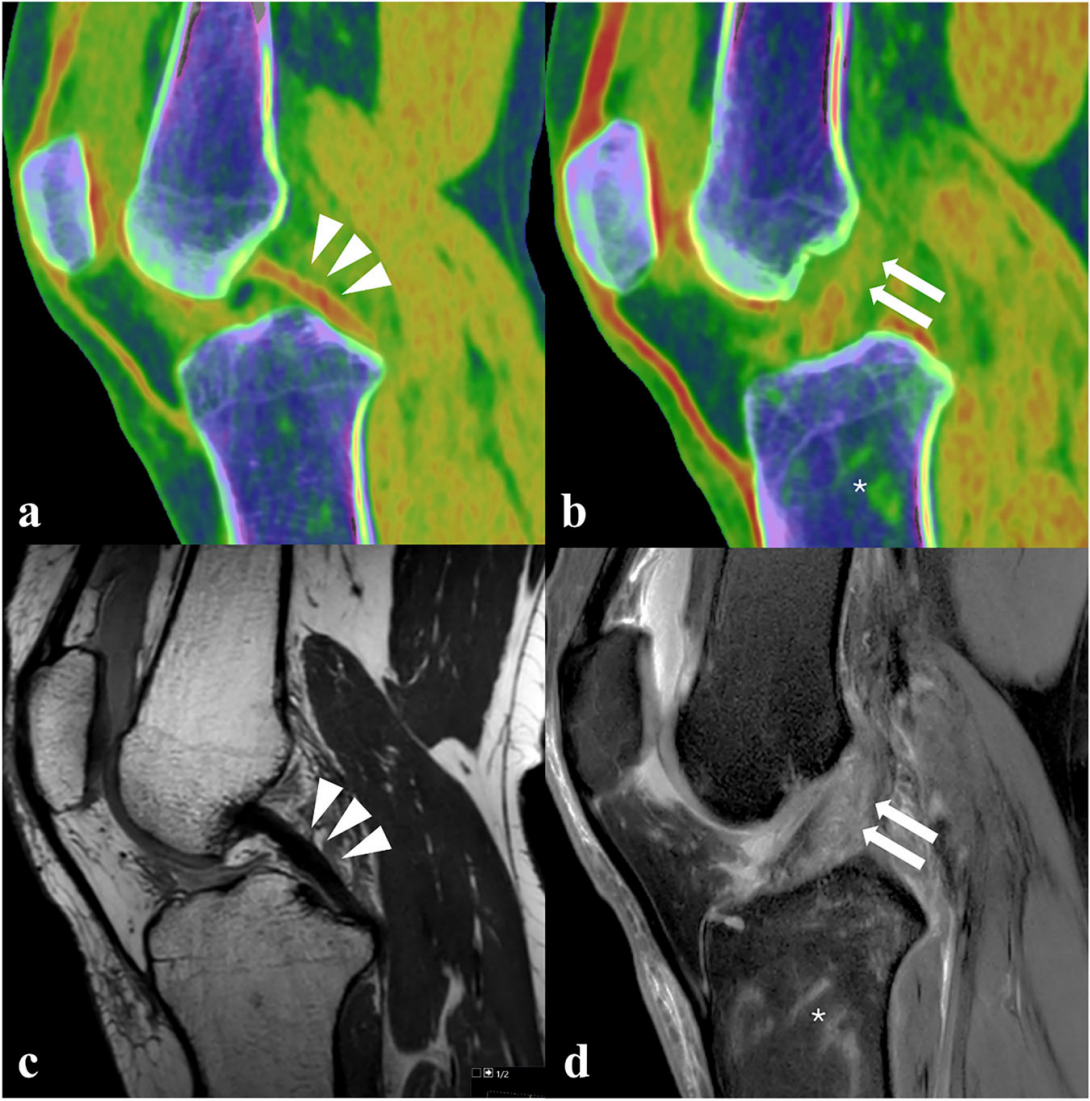
Imaging of cruciate ligament integrity and bone marrow edema using PCD-CT and MRI. **a** Color-coded VNCa image illustrating intact posterior cruciate ligament (PCL) (*red*, arrowheads). **b** Normal appearance of the PCL on the corresponding sagittal proton density-weighted MRI image (arrowheads). **c** Color-coded VNCa image allows the identification of ACL injury. The ACL injury is suspected based on the altered color patterns within the color-coding spectrum of the VNCa image, suggesting ligamentous fiber discontinuity (arrows). Note the focal bone marrow edema in the proximal tibial metaphysis (*, *green*). **d** Corresponding T2-weighted fat-saturated MRI image depicting ACL disruption (arrows) and bone marrow edema in the tibial metaphysis (*), correlating with PCD-CT findings

A PCD-CT scan was conducted to assess the extent of the osseous knee injury. Imaging was performed according to the protocol described above. CT images confirmed the presence of a Segond fracture at the lateral tibial plateau (Fig. 2b) and an additional fracture at the posterior side of the lateral tibial plateau with slight depression (Fig. 3a, b). Compared to the standard resolution images, comparable to conventional EID-CT (Fig. 3a), the UHR images (Fig. 3b) demonstrated enhanced fracture conspicuity and better delineation of the cortical discontinuity. Additionally, VNCa images were reconstructed from the spectral data to subjectively evaluate potential bone marrow involvement and assess possible soft tissue injury, due to the high clinical suspicion [12, 13]. Diffuse, intraosseous green color intensity was observed surrounding the posterolateral tibial plateau fracture (Figs. 2c and 3c), which was appreciated as associated with BME. Additionally, linear BME patterns in the lateral tibial metaphysis suggested a posttraumatic origin. No trabecular discontinuity was observed on UHR images in this region. Color-coded VNCa images demonstrated a blurred anterior cruciate ligament (ACL) with decreasing yellow-red color intensity, suspicious for a complete discontinuity (Fig. 4b), if compared to the intact, sharply defined yellow-red posterior cruciate ligament (PCL) (Fig. 4a). The clinical suspicion of an MCL injury could not be definitively confirmed on VNCa images, despite the presence of edematous (green color intensity) changes in the area. MRI, performed four days later, confirmed an intact PCL and complete avulsion of the ACL from its femoral origin with significant surrounding edema (Fig. 4d). The MRI findings also confirmed presence of the distinct linear BME pattern in the metaphysis that precisely matched the color-coded VNCa images from the PCD-CT scan (Fig. 2c, d). Also, a partial MCL injury was observed.

The patient was managed conservatively with a hinged knee brace, allowing full range of motion and weight-bearing as tolerated. Physiotherapy was initiated to restore joint mobility and regain muscle strength. Follow-up evaluation was scheduled for six weeks to monitor healing progression.

## Discussion

This technical note explores the implementation of UHR spectral PCD-CT for the integrated evaluation of fractures, bone marrow lesions, and soft tissue damage in acute knee trauma. Recent research has successfully demonstrated the use of CT in the diagnostic assessment of acute musculoskeletal imaging. While traditional CT imaging has been limited by inferior soft tissue contrast resolution in evaluating intra-articular soft tissue structures, advancements in CT technology, such as the introduction of DECT, have led to developments. These advancements offer the potential for tissue characterization, which until now has only been assessable using the reference standard, MRI. Various DE applications, such as VMIs at different energies, VNCa and collagen mapping reconstructions, with and without color-coding, have been used to characterize traumatic bone marrow lesions and other pathologies [11]. Gruenewald et al demonstrated significantly higher diagnostic accuracy for DECT in identifying cruciate ligament injuries in patients experiencing acute trauma compared to single-energy CT. The authors evaluated polychromatic grayscale images; no specific DE application was used to enhance the visualization of the ligaments [14]. In another study, DECT was used to detect ACL rupture, based on polychromatic grayscale images, color-coded VMIs at 80 keV, and electron density maps [5]. DECT provided high sensitivity (97.1%) and specificity (98.0%) for ACL rupture by applying quantitative measurements of CT-numbers in the images. However, the ability to reliably differentiate between partial and complete ruptures was not assessed. In a multi-reader diagnostic accuracy study, Booz et al evaluated color-coded VNCa images from DECT for detecting BME in patients with acute knee trauma [15]. The study demonstrated improved diagnostic accuracy for DECT compared to conventional imaging techniques. While existing studies explore the use of specific DE reconstruction techniques to enhance the visualization of specific anatomical structures, they do not provide a unified strategy for the comprehensive assessment of acute knee injury patterns using a single CT scan.

To our knowledge, this is the first description of the use of UHR spectral PCD-CT for the simultaneous assessment of fractures, BME, and cruciate ligament integrity in acute knee injuries with an integrated evaluation. The data were acquired using a scan mode optimized for comprehensive imaging of such injuries: the two PCDs of a dual source PCD-CT acquire spectral data at two different x-ray tube voltages to improve spectral separation.

The current case report demonstrates the potential added value of this integrated approach in clinical practice. However, as this is merely a feasibility case report, scientific validation must be obtained through a prospective clinical case series. For example, the inability to conclusively diagnose the partial MCL tear on VNCa images, despite its identification on MRI, underscores the need for a broader evidence base beyond a single case report. But also, the potential presence of mucoid degeneration may act as a confounding factor and should be considered in spectral image interpretation. To ensure reliable clinical implementation, direct comparisons between post-traumatic knee findings, including meniscal and chondral injuries, on PCD-CT and MRI should be conducted within a larger prospective cohort. This will facilitate the development of a standardized reference framework for PCD-CT and VNCa images, similar to the well-established benchmarks used in MRI.

The volume Computed Tomography Dose Index (CTDIvol) in this case was approximately 7.59 mGy. This is in line with reported dose values for CT knee examinations, which range between 9.1 mGy for standard CT and 2.5 mGy for dose-optimized tin-filtered CT [16]. The corresponding dose-length product (DLP) was 167 mGy·cm, and the estimated effective dose was approximately 0.16 mSv. Given that the quantum peak protocol provides additional diagnostic information, the total exposure of 0.16 mSv seems justifiable. Future research should focus on possible further dose optimization.

In summary, based on this preliminary experience, this advanced imaging technique appears to enhance diagnostic accuracy by simultaneously providing detailed structural and compositional information of both bone and soft tissue, which may be valuable as early triage in the acute phase. However, further studies in larger cohorts are needed to determine whether this integrated approach truly offers clinical added value in acute knee trauma.

## Supplementary information

**Additional file 1**: **Table S1**. Scan and reconstruction parameters for post-traumatic knee imaging using PCD-CT.

## Data Availability

The datasets generated and/or analyzed during the current study are available from the corresponding author upon reasonable request.

## Abbreviations

ACL: Anterior cruciate ligament
BME: Bone marrow edema
DECT: Dual-energy CT
EID: Energy-integrating detectors
MRI: Magnetic resonance imaging
MCL: Medial collateral ligament
PCD-CT: Photon-counting detector CT
PCL: Posterior cruciate ligament
SPP: Spectral post-processing
UHR: Ultra-high resolution
VNCa: Virtual non-calcium
