# Supplementary material for: The emerging role of photon-counting detector CT: primary experience on the integrated assessment of acute knee injuries
Source: Eur Radiol Exp. 2025 Aug 9;9:71. doi: 10.1186/s41747-025-00616-8 (PMC12335411; doi:10.1186/s41747-025-00616-8)
Supplement: Supplementary file 1 — Additional file 1: Table S1. Scan and reconstruction parameters for post-traumatic knee imaging using PCD-CT. [file 41747_2025_616_MOESM1_ESM.pdf]

The Emerging Role of Photon-counting Detector CT: Primary Experience on the Integrated Assessment of Acute Knee Injuries

ELECTRONIC SUPPLEMENTARY MATERIAL

Table S1. Scan and reconstruction parameters for post-traumatic knee imaging using PCD-CT

**Scan parameters**		
Scan protocol	Quantum Peak	
X-ray tube voltages	70 kV / Sn 150 kV	
Detector collimation	96x0.2 mm	
Gantry rotation time	0.25 s	
Pitch	0.8	
IQ level	75	
**Image reconstruction parameters**		
	UHR images	Spectral SPP images
Kernel	Br80	Qr44
QIR level	3	3
Reconstructed slice-thickness	0.2 mm	0.8 mm
Increment	0.2 mm	0.5 mm
